# Early imaging differentiation of children with acute hematologic malignancies that present in pancytopenia: a retrospective study

**DOI:** 10.3389/fonc.2026.1775816

**Published:** 2026-05-28

**Authors:** Junya Ma, Ye Wang, Ye Xu, Yu He, Xinyu Zhang, Jun Li, Sijie Gao, Wei Ma, Dajian Dai

**Affiliations:** 1 Department of Radiology, Children’s Hospital of Chongqing Medical University, National Clinical Research Center for Children and Adolescents' Health and Diseases, Ministry of Education Key Laboratory of Child Development and Disorders, Chongqing Key Laboratory of Child Rare Diseases in Infection and Immunity, Chongqing, China; 2 Department of Radiology, People’s Hospital of Chongqing Yubei District, Chongqing, China

**Keywords:** computed tomography, pancytopenia, hematologic malignancies, pediatric, imaging

## Abstract

**Objectives:**

This retrospective cohort study aimed to identify potential differentiation indicators on thoracic and abdominal computed tomography (CT) among three hematologic malignancies characterized by acute pancytopenia in children.

**Methods:**

This study was conducted at the Children’s Hospital of Chongqing Medical University, Chongqing, China. A total of 133 children with acute pancytopenia from August 2016 to November 2024 were enrolled, including 23 with malignancy-associated hemophagocytic lymphohistiocytosis (M-HLH), 58 with acute leukemia (AL), and 52 with Epstein–Barr virus-associated HLH (EBV-HLH). The differences in thoracic and abdominal CT features were statistically analyzed.

**Results:**

Chest CT showed significant intergroup differences in pulmonary lesions, thickening of the bronchiovascular bundle, and pleural effusion. Mediastinal largest lymph nodes (LLNs) in M-HLH were significantly larger, with receiver operating characteristic (ROC)-derived cutoff values of 11.45 mm (M-HLH vs. AL, sensitivity 65.2%, specificity 98.2%) and 11.60 mm (M-HLH vs. EBV-HLH, sensitivity 65.2%, specificity 100%), predominantly located in the retroinnominate space. Abdominal CT revealed significant differences in widened periportal space, ascites, splenomegaly, and intrarenal lesions. Abdominal LLNs in M-HLH were larger, with cutoff values of 9.3 mm (M-HLH vs. AL, sensitivity 72.7%, specificity 77.2%) and 9.60 mm (M-HLH vs. EBV-HLH, sensitivity 72.7%, specificity 75.0%).

**Conclusions:**

Thoracic and abdominal CT manifestations are usually different among pediatric hematologic malignancies that present in acute pancytopenia, providing valuable supportive clues for differential diagnosis.

## 1 Introduction

Pancytopenia is a common finding in clinical pediatric hematology, and hematologic malignancies are among the main causes (1). Of the malignancies, hypoproliferative acute leukemias (AL) and hemophagocytic lymphohistiocytosis (HLH) are two major contributors. Despite heterogeneous prognoses, these diseases generally have unfavorable outcomes, emphasizing the critical importance of early and accurate diagnosis for guiding appropriate treatment (1–3). HLH is more prevalent than previously reported; over a 5-year period, our institution diagnosed 311 pediatric cases, with an average of 54 cases annually. Pathogenetically, excessive activation and proliferation of T cells and the resulting severe systemic inflammation are currently thought to be the culprit (4). HLH contains a congenital and secondary forms, with diagnosis followed the HLH-2004 guidelines. Secondary HLH requires identification of underlying causes, including infections, tumors, or autoimmune disorders. Epstein–Barr virus-associated hemophagocytic lymphohistiocytosis (EBV-HLH) and malignancy-associated HLH (M-HLH) are comparably common in children despite variable incidences reported (3, 5, 6). Patients with M-HLH, mostly lymphoma or leukemia, have symptoms similar to those of EBV-HLH but different treatment and prognoses (7). Hypoproliferative AL, accounting for 8.69-11.02% of acute lymphoblastic leukemia cases, also presents with pancytopenia and is characterized by rapid disease progression, accompanied by overt anemia, hemorrhage, hepatosplenomegaly, and lymphadenopathy (8–10). These clinical overlaps make differential diagnosis between EBV-HLH, M-HLH, and AL challenging (2, 8, 10, 11). Imaging examinations, offer rapid and non-invasive assessment of organ involvement, facilitating etiological investigation and supporting clinical decision-making (12, 13). However, the literature on imaging differentiation of hematologic malignancies presenting with pancytopenia remains limited, and significant overlap in imaging findings further complicates diagnosis (12–16). Therefore, this retrospective study aimed to analyze thoracic and abdominal computed tomography CT manifestations of pediatric patients with EBV-HLH, M-HLH, and AL presenting with pancytopenia, with the goal of identifying imaging features that may provide supplementary information for differential diagnosis when clinical and laboratory assessments are inconclusive.

## 2 Methods

### 2.1 General information

This study was performed in compliance with the protocol approved by our institutional research ethics board (protocol number: 2019-87), and informed consent was waived. The study was conducted at the Children’s Hospital of Chongqing Medical University, a tertiary referral center in Chongqing, China. Clinical and imaging data of children admitted with pancytopenia between August 2016 and November 2024 were retrieved from the hospital electronic medical record system and picture archiving and communication system.

**Figure 1:**
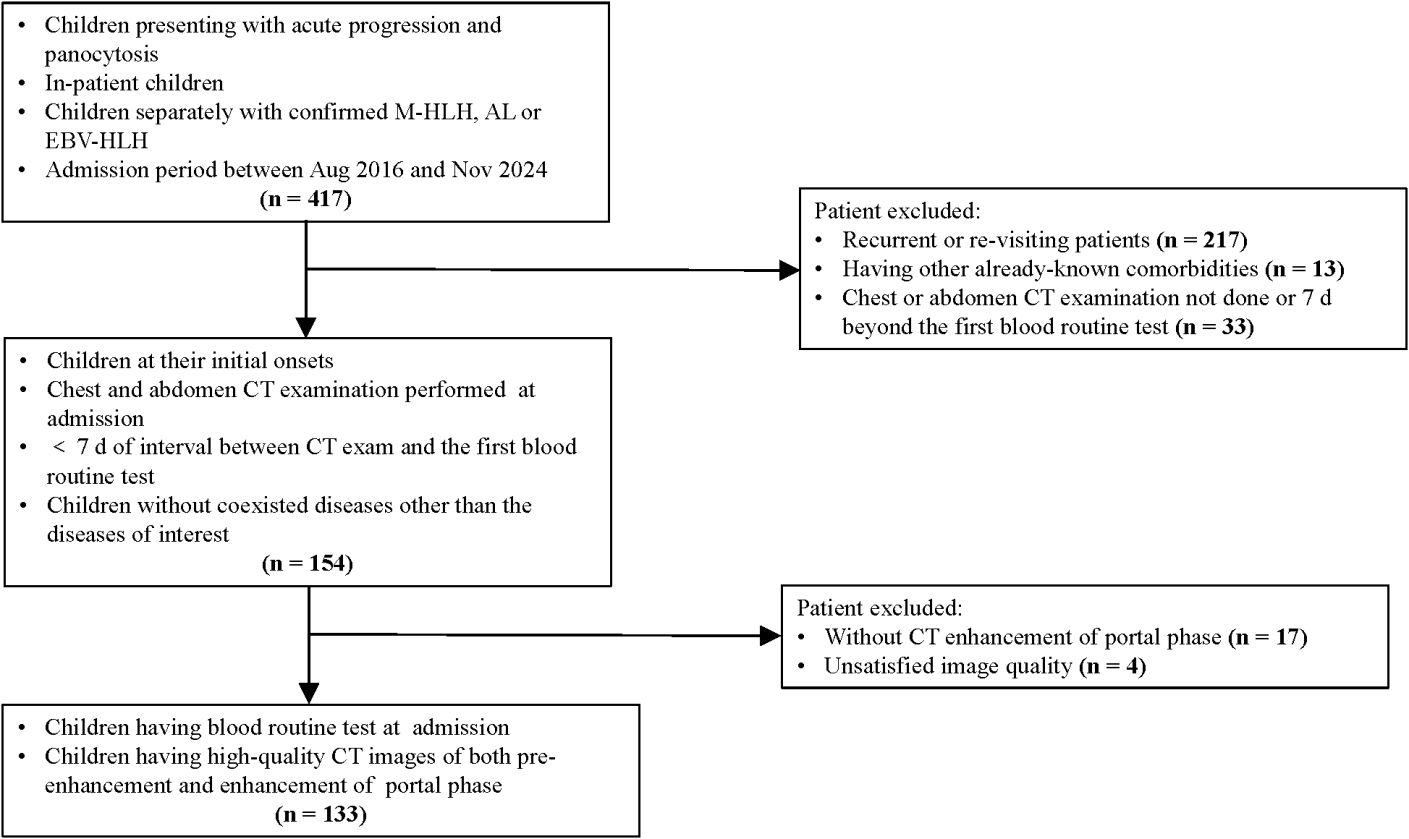
Flowchart of study patients. M-HLH, malignancy associated hemophagocytic lymphohistiocytosis; AL, acute leukemia; EBV-HLH, Epstein–Barr virus associated hemophagocytic lymphohistiocytosis.

The diagnostic criteria for pancytopenia in children were defined as follows: white blood cell count< 4.0 ×10^9^/L; red blood cell (RBC) count below the age-specific lower limit of normal based on Quest Diagnostics pediatric reference ranges; platelet count< 100×10^9^/L. The diagnosis of secondary HLH followed the HLH-2004 guidelines, meeting at least 5 of the 8 items. The diagnosis of EBV infection met at least one of the following criteria: *(a)* serological antibody test suggestive of primary acute EBV infection or active infection; *(b)* positive EBV-DNA in serum, bone marrow or lymph nodes by molecular methods, including polymerase chain reaction (PCR), *in situ* hybridization or/and southern blot hybridization (1, 2). The diagnosis of leukemia or lymphoma was made by surgical biopsy of bone marrow or tumor tissue. The study inclusion criteria were as follows: *(a)* the diagnosis of M-HLH, AL or EBV-HLH was made for the first time; *(b)* thoracic or abdominal CT scans contained both nonenhancement and enhancement. The exclusion criteria were as follows: *(a)* patients with coexisting acute or chronic conditions, such as congenital heart disease, trauma, systemic infections and other malignancies; *(b)* TIFF > 7 days; *(c)* incomplete test results or unsatisfactory image quality. (**Figure 1**). **Figure 1** provides a detailed illustration of participant flow through enrollment, inclusion, exclusion, and analysis.

Data on demographics, disease subtypes, first blood routine test (BRT), time interval between the first BRT and the first CT examination (TIFF), short diameter of the largest lymph node (LLN) in the thoracic cavity, and the axilla, abdominal cavity and groin were measured and collected. Positive rates of several important indicators were also evaluated, including pulmonary lesions, intrarenal focal lesions (IFLs) of kidney, thickening of the bronchiovascular bundle (TBB), pleural effusion, widened periportal space (WPS), ascites, hepatomegaly, and splenomegaly (3, 4).

### 2.2 CT scanning parameters

All CT scans were performed using a LightSpeed VCT 64-slice CT scanner (GE LightSpeed, Waukesha, WI). Scanning parameters are detailed in **Supplementary Table 1**.

### 2.3 Image analysis

Image analysis was performed independently by two radiologists (J.M. and S.G.) with more than 10 years of experience in pediatric imaging, who were blinded to patient diagnosis and grouping. Prior to analysis, the radiologists received standardized training to ensure consistency in measurement methods and definition interpretation. All measurements were performed on contrast-enhanced images. Discrepancies were resolved by consensus with a third radiologist (Y.X.) with more than 30 years of imaging experience. To minimize bias, the radiologists were blinded to all clinical information except the CT images, and the order of image review was randomized.

**Figure 2:**
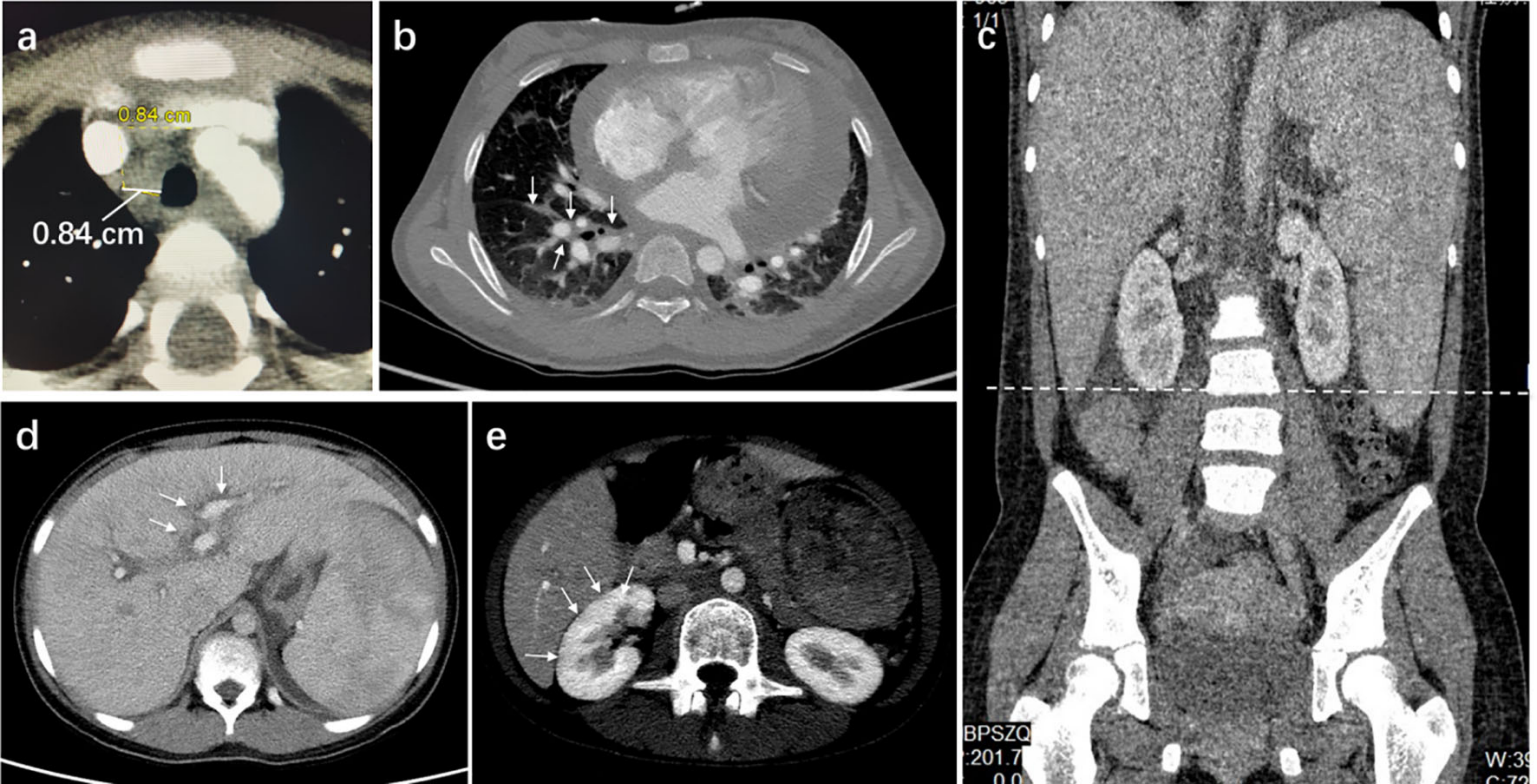
measurement and definition of several major imaging variables. **(a)** Measurement of the short diameter of the largest lymph node. **(b)** Thickening of the bronchiovascular bundle (white arrows). **(c)** Hepatomegaly: the lower border of the liver exceeds the lower border of the right kidney (dashed line) or is >2 cm below the costal margin. Splenomegaly: the lower border of the spleen exceeds any of the following three levels: the lower border of the liver, the lower border of the left kidney, or the costal margin. **(d)** Widened periportal space (white arrows). **(e)** Intrarenal lesions with markedly low attenuation compared to the normal kidney (white arrows).

The imaging terms and variables involved in this study were defined in **Table 1** and **Figure 2**.

**Table 1:** Definitions of key imaging terms.

Imaging terms	Definition
TBB	Slightly enhanced abnormal soft tissue distributed from the hilum toward the large branches of the basilar bronchiovascular bundles
Hepatomegaly	Lower border of the liver exceeding the lower border of the right kidney or 2 cm under the costal margin
Splenomegaly	Lower border of the spleen exceeding one of three levels: the lower border of the liver, the lower border of the left kidney, or the costal margin
WPS	Non-enhanced hypoattenuation strips encircling portal veins, extending down to at least 3-grade intrahepatic branches
IFL of renal	Intrarenal lesions with markedly low attenuation compared to normal renal parenchyma

TBB, thickening of the bronchiovascular bundle; WPS, widened periportal space; IFL, intrarenal focal lesions.

### 2.4 Sample size determination

This was a retrospective study, and no *a priori* sample size calculation was performed. The sample size was determined by the number of eligible patients who met the inclusion criteria during the study period (August 2016 to November 2024). All consecutive patients who fulfilled the criteria were enrolled.

## 3 Statistical analysis

Statistical analyses were conducted with IBM SPSS Statistics 20.0 (Chicago, IL, USA). The Shapiro–Wilk test was employed to test whether the data were normally distributed. If yes, data are presented as the mean ± standard deviation ( $\bar{x}\pm \text{s}$), and a variance analysis was used; if not, data are expressed as the median and upper and lower quartiles *[M (Q1, Q3)]*, and the Kruskal–Wallis rank sum test was adopted. For *P* values< 0.05, the Mann–Whitney U test was used for pairwise comparisons. Count data were expressed as ratios or percentages (%), and differences between groups were analyzed using the chi-square test or Fisher’s exact test. A receiver operating characteristic (ROC) curve was used for meaningful continuous variables to determine the sensitivity, specificity and cutoff value. A p value< 0.05 was considered statistically significant. There were no missing data for the primary imaging and clinical variables included in this study, as cases with incomplete test results were excluded during the screening process (**Figure 1**).

## 4 Results

### 4.1 Clinical characteristics

#### 4.1.1 Demographic data

A total of 133 pediatric patients with pancytopenia were enrolled in this retrospective study, including 23 cases in Group 1 (malignancy-associated hemophagocytic lymphohistiocytosis, M-HLH), 58 cases in Group 2 (acute leukemia, AL), and 52 cases in Group 3 (Epstein–Barr virus-associated HLH, EBV-HLH) (**Table 2**). No significant differences were found among the groups for gender (p = 0.92). However, age distribution differed significantly (p = 0.001), with children in Group 1 (median age 9.2 years) being significantly older than those in Group 2 (median age 4.6 years) and Group 3 (median age 4.2 years) (p< 0.001) (**Table 2**).

**Table 2:** General data of the patients.

Clinical variables	Group 1 (M-HLH, n=23)	Group 2 (AL, n=58)	Group 3 (EBV-HLH, n=52)	*P* value
Sex				
Male	11	31	26	0.92
Female	12	27	26	
Age (y)	9.2 (6.3, 11.6)	4.6 (2.9, 8.3)	4.2 (2.2, 7.0)	0.001
Blood cell count				
Erythrocytes (× 10^12^/L)	3.2 (2.9, 4.0)	2.6 (2.2, 3.1)	3.4 (3.1, 3.8)	<0.001
Leukocytes (× 10^9^/L)	2.9 (1.1, 7.6)	2.9 (2.1, 3.7)	2.5 (1.4, 3.0)	0.01
Thrombocytes (× 10^9^/L)	76 (52, 106)	35 (19, 64)	44 (29, 66)	<0.001
TIFF (d)	2 (1, 2)	2 (1, 3)	2 (1, 3)	0.86

The values in the table are expressed as the median and interquartile range.

BRT, blood routine test; TIFF, time interval between the first CT examination and the first blood routine test; M-HLH, malignancy associated hemophagocytic lymphohistiocytosis; AL, acute leukemia; EBV-HLH, Epstein–Barr virus associated hemophagocytic lymphohistiocytosis.

#### 4.1.2 Composition of disease subtypes

Group 1 (M-HLH) included 6 children with non-Hodgkin’s lymphoma, 5 children with anaplastic large cell lymphoma, 4 with B-lymphocytic leukemia, 2 with ALK-positive anaplastic large cell lymphoma, 3 with EBV-associated NK/T-cell lymphoma, 1 with acute early pre-T-lymphoblastic leukemia, 1 with intranodal NK/T-cell lymphoma and 1 with T-cell non-Hodgkin’s lymphoma. Group 2 (AL) included 38 children with acute common B-lymphoblastic leukemia, 8 children with pre-B lymphoblastic leukemia, 4 with acute myeloid leukemia, 4 with acute promyelocytic leukemia, 2 with acute T-lymphoblastic leukemia, 1 with acute monocytic leukemia and 1 with acute pre-T-lymphoblastic leukemia. All 52 children in Group 3 had EBV-HLH.

#### 4.1.3 Blood routine test results

All patients presented with pancytopenia at admission. Significant differences were observed in leukocytes between Group 2 and Group 3 (p = 0.01). Notably, Group 2 exhibited significantly lower erythrocytes and platelet counts compared to both Group 1 and Group 3 (all p< 0.001), with statistical differences confirmed by pairwise comparisons (**Table 2**).

#### 4.1.4 Time interval between first BRT and CT

There were no significant differences among the groups (p = 0.86) (**Table 2**).

### 4.2 Imaging features

#### 4.2.1 Chest imaging

The imaging features and statistical results of thoracic CT are presented in **Table 3**.

**Table 3:** Imaging data analysis of thoracic CT.

Imaging variables	Group 1 (M-HLH, n=23)	Group 2 (AL, n=58)	Group 3 (EBV-HLH, n=52)	*P* value
Pulmonary lesions (case, %)	11 (47.8%)	28, (49.1%)	43, (82.7%)	< 0.001
TBB (case, %)	6 (26.1%)	3 (5.3%)	0 (0%)	< 0.001
Pleural effusion (case, %)	14 (60.9%)	8 (14.0%)	35 (67.3%)	< 0.001
Short diameter of mediastinal LLN (mm)	13.9 (6.7, 15.8)	5.1(4.0, 6.75)	5.6 (4.8, 7.73)	< 0.001
Site of mediastinal LLN (site, case,%)				0.03
Retroinnominate space	14 (60.9%)	19 (32.7%)	19 (36.5%)	
Subcarinal space	8 (34.8%)	38 (65.5%)	33 (63.5%)	
Peri-aortic arch	1 (4.3%)	1 (1.7%)	0 (0%)	
Short diameter of axillary LLN (mm)	7.2 (3.2, 11.0)	5.6 (4.1, 6.3)	4.6 (4.0, 5.7)	0.04

The values in the table are expressed as the median and interquartile range.

TBB, thickening of the bronchiovascular bundle; LLN, largest lymph node; M-HLH, malignancy associated hemophagocytic lymphohistiocytosis; AL, acute leukemia; EBV-HLH, Epstein–Barr virus associated hemophagocytic lymphohistiocytosis.

##### 4.2.1.1 Pulmonary lesions

Forms of pulmonary lesions included consolidation, ground-glass opacities, atelectasis, thickening of the bronchiovascular bundle, nodules and dense band-like shadows. The positive rates of pulmonary lesions were 47.8%, 49.1% and 82.7% for Groups 1, 2 and 3, respectively, with Group 3 having significantly higher rates than the other groups (all p< 0.05, **Table 3**).

##### 4.2.1.2 TBB

The positive rates of TBB were 26.1%, 5.3% and 0.0% for Groups 1, 2 and 3, respectively, with the rate of Group 1 being significantly higher than that of Group 2 (p = 0.01) and Group 3 (p*<* 0.001).

##### 4.2.1.3 Pleural effusion

The positive rates of pleural effusion were 60.9%, 14.0% and 67.3% for Groups 1, 2 and 3, respectively, with Group 2 having a significantly lower rate than Group 1 and Group 3 (p< 0.001 for both).

##### 4.2.1.4 Mediastinal LLN

**Figure 3:**
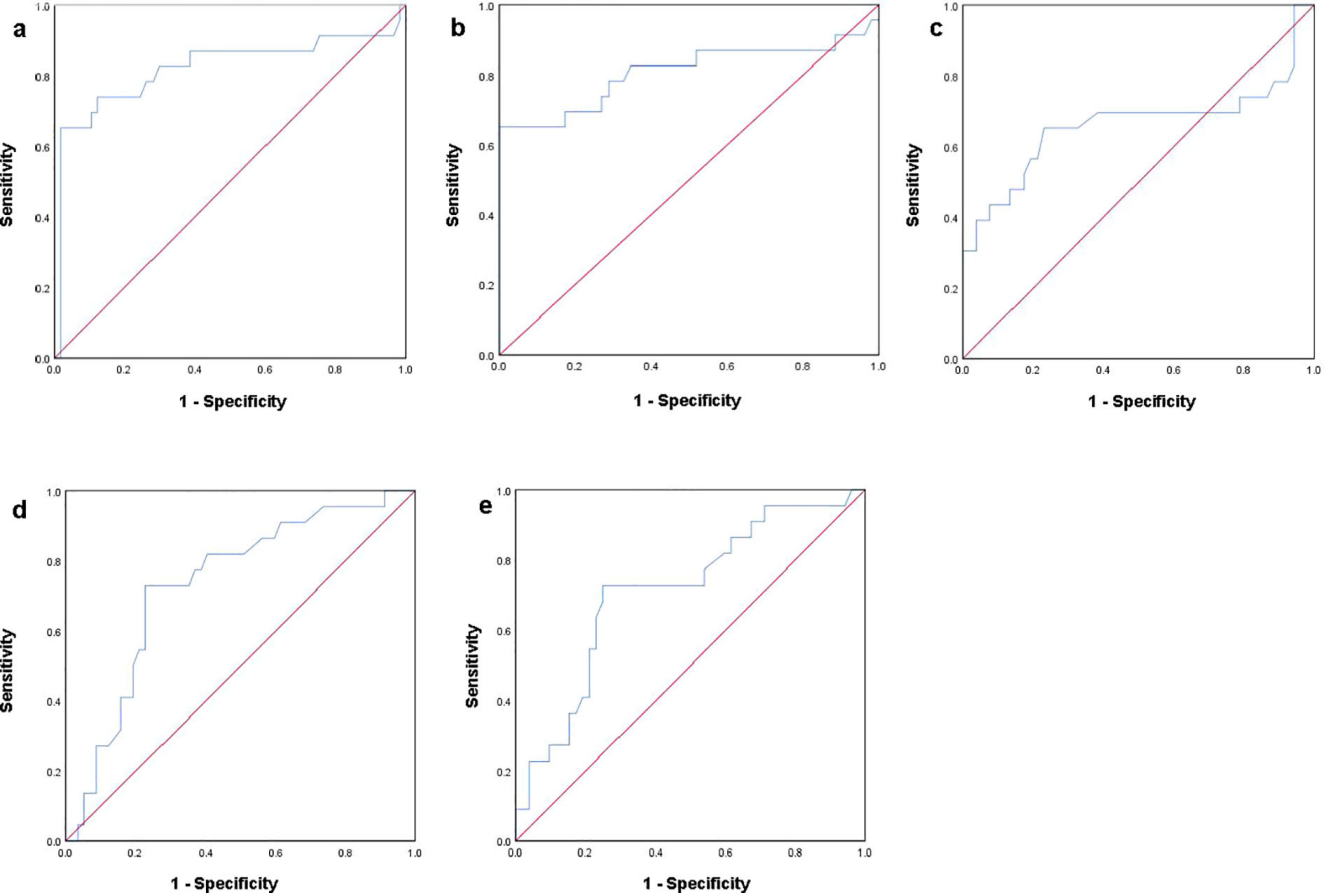
ROC curve analysis of the short diameter of mediastinal, axillary and celiac LLNs. **(a)** ROC curve for the short diameter of mediastinal LLNs (Group 1 vs. Group 2): The AUC was 0.821 (P< 0.001, 95%CI: 0.693–0.949), with an optimal cutoff value of 11.45 mm (sensitivity: 65.2%, specificity: 98.2%); **(b)** ROC curve for the short diameter of mediastinal LLNs (Group 1 vs. Group 3): The AUC was 0.807 (P< 0.001, 95% CI: 0.671–0.943), with an optimal cutoff value of 11.60 mm (sensitivity: 65.2%, specificity: 100%); **(c)** ROC curve for the short diameter of axillary LLNs (Group 1 vs. Group 3): The AUC was 0.661 (P = 0.027, 95% CI: 0.496–0.825), with an optimal cutoff value of 5.95 mm (sensitivity: 65.2%, specificity: 76.9%); **(d)** ROC curve for the short diameter of celiac LLNs (Group 1 vs. Group 2): The AUC was 0.733 (P = 0.001, 95% CI: 0.612–0.854), with an optimal cutoff value of 9.3 mm (sensitivity: 72.7%, specificity: 77.2%); **(e)** ROC curve for the short diameter of celiac LLNs (Group 1 vs. Group 3): The AUC was 0.712 (P = 0.004, 95% CI: 0.581–0.842), with an optimal cutoff value of 9.60 mm (sensitivity: 72.7%, specificity: 75.0%). ROC, receiver operating characteristic; LLN, largest lymph node; AUC, area under the curve; CI, confidence interval.

The short diameter of mediastinal LLNs in Group 1 (median 13.9 mm) was significantly larger than that in Group 2 (median 5.1 mm) and Group 3 (median 5.6 mm) (p< 0.001 for both). ROC curve analysis showed an area under the curve (AUC) of 0.82 (95% CI: 0.693-0.949, p<0.001) for distinguishing M-HLH (Group 1) from AL (Group 2), with an optimal cutoff value of 11.45 mm (sensitivity 65.2%, specificity 98.2%). For distinguishing M-HLH from EBV-HLH (Group 3), the AUC was 0.807 (95% CI: 0.671-0.943, p<0.001), with a cutoff value of 11.60 mm (sensitivity 65.2%, specificity 100%) (**Figures 3a, b**). Regarding distribution, mediastinal LLNs in Group 1 were predominantly located in the retroinnominate space (14 cases, 60.9%), while Group 2 and Group 3 showed a higher prevalence in the subcarinal space (38 and 33 cases, 65.5% and 63.5%, respectively) (p = 0.03).

##### 4.2.1.5 Axillary LLN

The short diameter of axillary LLNs in Group 1 (median 7.2 mm) was significantly larger than that in Group 2 (median 5.6 mm) and Group 3 (median 4.6 mm) (p = 0.04). ROC curve analysis for distinguishing M-HLH (Group 1) from EBV-HLH (Group 3) yielded an AUC of 0.661 (95% CI: 0.496-0.825, p=0.03), with a cutoff value of 5.95 mm (sensitivity 65.2%, specificity 76.9%) (**Figure 3c**).

#### 4.2.2 Abdominal imaging

The imaging features and statistical results of abdominal CT are presented in **Table 4**.

**Table 4:** Imaging data analysis of abdominal CT.

Imaging variables	Group 1 (M-HLH, n=23)	Group 2 (AL, n=58)	Group 3 (EBV-HLH, n=52)	*P* value
Hepatomegaly (case, %)	23 (100%)	53 (91.4%)	51 (98.1%)	0.17
WPS (case, %)	12 (54.5%)	9 (15.5%)	41 (78.8%)	< 0.001
Splenomegaly (case, %)	15 (68.1%)	25 (43.1%)	49 (94.2%)	< 0.001
Ascites (case, %)	16 (72.7%)	9 (15.5%)	37 (71.2%)	< 0.001
IFL (case, %)	3 (13.6%)	11 (19.0%)	1 (1.9%)	0.02
SDCLLN (mm)	10.7 (6.1, 13.3)	5.3 (4.1, 6.8)	5.6 (3.4, 9.8)	0.003
Site of celiac LLN (site, case,%)				0.01
Peri-aorta	13 (56.5%)	35 (60.3%)	28 (53.8%)	
porta hepatis	7 (30.5%)	13 (22.4%)	24 (46.2%)	
iliac fossa	3 (13.0%)	7 (12.1%)	0 (0%)	
mesentery	0 (0%)	3 (5.2%)	0 (0%)	
Short diameter of inguinal LLN (mm)	5.3 (3.8, 7.7)	4.0 (3.3, 4.9)	4.2 (3.5, 5.3)	0.07

The values in the table are expressed as the median and interquartile range.

WPS, widened periportal space; IFL, intrarenal focal lesion; SDCLLN, short diameter of the largest celiac lymph node; LLN, largest lymph node; M-HLH, malignancy associated hemophagocytic lymphohistiocytosis; AL, acute leukemia; EBV-HLH, Epstein–Barr virus associated hemophagocytic lymphohistiocytosis.

##### 4.2.2.1 Hepatomegaly

The positive rates of hepatomegaly were 100%, 91.4% and 98.1% for Groups 1, 2 and 3, respectively, without a significant difference among them (p = 0.17).

##### 4.2.2.2 Widened periportal space

The positive rates of WPS were 54.5%, 15.5% and 78.8% for Groups 1, 2 and 3, respectively, with a significant difference between Groups 1 and 2 (p = 0.001), Groups 1 and 3 (p = 0.049), and Groups 2 and 3 (p< 0.001).

##### 4.2.2.3 Splenomegaly

The positive rates of splenomegaly were 68.1%, 43.1% and 94.2% for Groups 1, 2 and 3, respectively, with a significantly higher rate in Group 3 than in Group 1 (p = 0.006) and Group 2 (p< 0.001).

##### 4.2.2.4 Ascites

The positive rates of ascites were 72.7%, 15.5% and 71.2% for Groups 1, 2 and 3, respectively, with a significantly lower rate in Group 2 than in the other two groups (p< 0.001 for both).

##### 4.2.2.5 Intrarenal focal lesions

The positive rates of IFLs of kidney were 13.6%, 19.0% and 1.9% for Groups 1, 2 and 3, respectively, with a significantly lower rate in Group 3 than in Group 2 (p = 0.005).

##### 4.2.2.6 Celiac LLN

The size and distribution of celiac LLNs are summarized in **Table 4**. The short diameter of the celiac LLN in Group 1 (median 10.7 mm) was significantly larger than that in Group 2 (median 5.3 mm, p = 0.001) and Group 3 (median 5.6 mm, p = 0.004). ROC curve analysis for distinguishing Group 1(M-HLH) from Group 2 (AL) showed an AUC of 0.733 (95% CI: 0.612-0.854, p=0.001), with a cutoff value of 9.3 mm (sensitivity 72.7%, specificity 77.2%) (**Figure 3d**). For distinguishing Group 1 (M-HLH) from Group 3 (EBV-HLH), the AUC was 0.712 (95% CI: 0.581-0.842, p=0.004), with a cutoff value of 9.60 mm (sensitivity 72.7%, specificity 75.0%) (**Figure 3e**).

Regarding the distribution of celiac LLNs, the statistical analysis showed a significant difference between Groups 2 and 3 in both the perihepatic portal area (22.4% vs. 46.2%, p = 0.012) and the iliac fossa (12.1% vs. 0%, p = 0.01) (**Table 4**).

##### 4.2.2.7 Inguinal LLN

No significant intergroup difference was observed in the short diameter of inguinal LLNs (p = 0.07).

**Figure 4:**
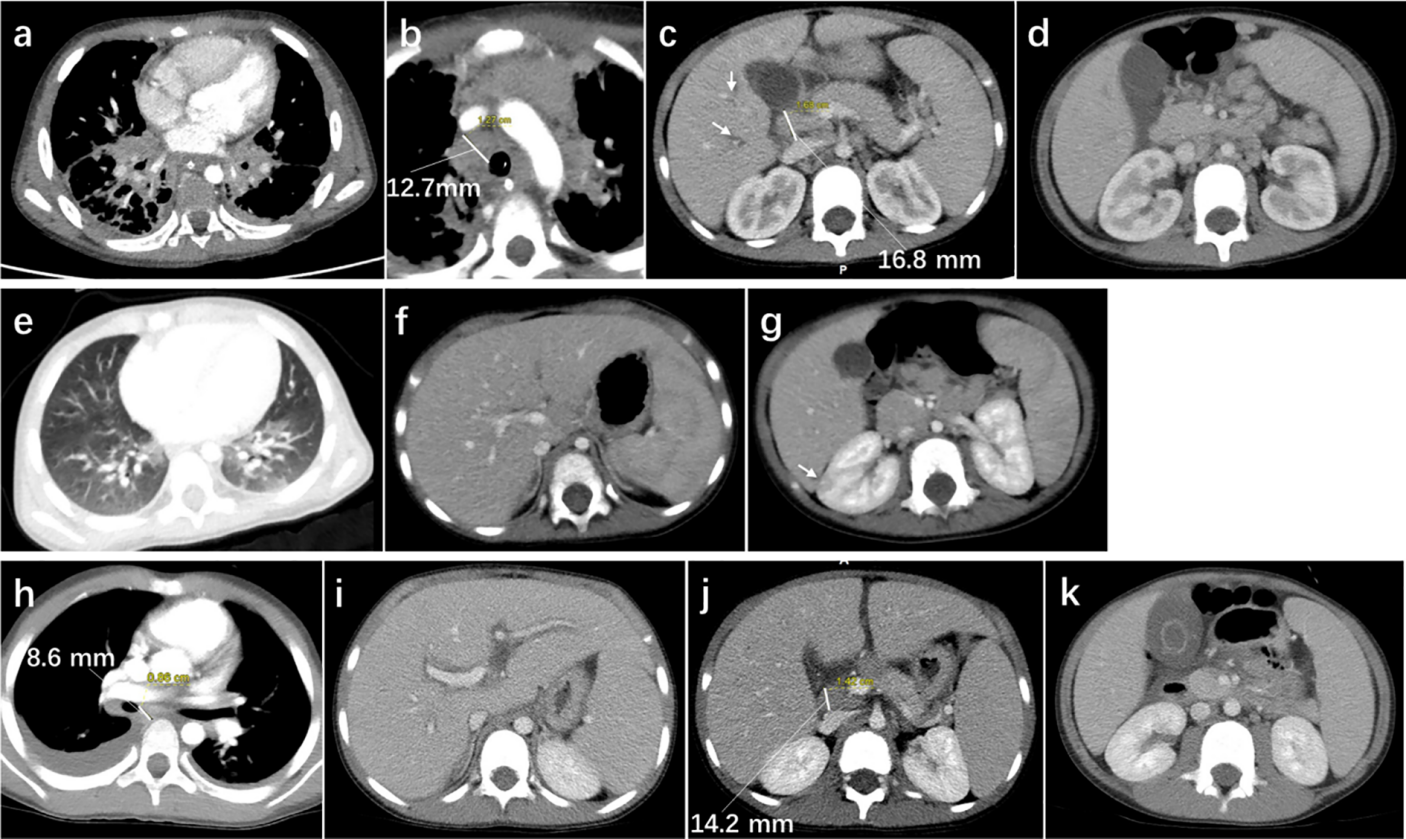
Representative CT images of the three groups. **(a–d)** a boy aged 9 years and 3 months was diagnosed with anaplastic large cell lymphoma associated with hemophagocytic lymphohistiocytosis (Group 1). Thoracic and abdominal CT identified thickening of bronchiovascular bundle, the mediastinal largest lymph node at retroinnominate space (with a short diameter of 12.7 mm), the celiac largest lymph node at perihepatic portal area space (with a short diameter of 16.8 mm). In addition, signs of hepatomegaly, splenomegaly and widened periportal space (white arrows) were also observed. **(e–g)** another boy, aged 2 years and 10 months, was diagnosed with acute common B-lymphoblastic leukemia (Group 2). CT showed no effusion in the pleural and peritoneal cavities, no widened periportal space and only a small region of interstitial lesions in the left lower lung, perhaps suggesting leukemia infiltration. In addition, signs of hepatomegaly, splenomegaly and intrarenal focal lesion (white arrows) were observed. **(h–k)** a third boy, aged 7 years, was diagnosed with EBV associated hemophagocytic lymphohistiocytosis (Group 3). CT showed hydrothorax, ascites and the mediastinal largest lymph node at sub-carina space (with a short diameter of 8.6 mm). In addition, signs of hepatomegaly, splenomegaly, widened periportal space and no lesions within both kidney were also observed as representative findings of Group 3. However, the largest node with a short diameter of 14.2 mm were not common for children in Group 3.

Representative CT images of the three groups are shown in **Figure 4**.

## 5 Discussion

M-HLH, hypoproliferative AL and EBV-HLH all often present with acute symptoms, especially in severely affected children. It is not always easy to reach a correct diagnosis because it depends on doctors’ decisions, valid tests, effective biopsies and skilled operations. Imaging examinations at the early stage of disease may provide supportive visual information on organ involvement and severity of illness, which may aid in the differential diagnostic process when combined with clinical data (11, 17, 18).

In this study, we found that all enrolled patients presented with pancytopenia, a core clinical feature of the three malignancies, yet significant intergroup hematological disparities were noted. AL (Group 2) exhibited the lowest platelet count and erythrocytes, with significant differences compared to M-HLH (Group 1) and EBV-HLH (Group 3). Leukocytes was higher in AL than in EBV-HLH. These discrepancies stem from distinct pathogeneses: AL is characterized by bone marrow infiltration and suppression of normal hematopoiesis, resulting in the most severe cytopenias of the three, whereas HLH subtypes are driven by systemic inflammation with relatively milder impacts on erythropoiesis and thrombopoiesis (18).

Pulmonary involvement was common across all three groups, but the highest incidence was observed in EBV-HLH (82.7%), consistent with previous reports indicating a high prevalence of lung involvement in HLH (12, 15). Notably, TBB was most frequent in M-HLH (26.1%), with a significantly lower incidence in AL (5.3%) and no cases in EBV-HLH. Since lymphoma is the predominant subtype of M-HLH in this cohort, TBB may be attributed to lymphoma cell infiltration along the bronchiovascular bundles. Thus, the presence of TBB may be more supportive of M-HLH than EBV-HLH, which aligns with the findings of Fitzgerald et al. (15, 19). Furthermore, we observed the occurrence of TBB and found a higher percentage in the M-HLH group (26.1%) than in the other groups (5.3% and 0.0%).

Lymph node involvement is a key imaging feature of these hematologic malignancies. Our results showed that the short diameter of mediastinal LLNs was significantly larger in M-HLH than in AL and EBV-HLH. ROC curve analysis identified cutoff values of 11.45 mm and 11.60 mm for distinguishing M-HLH from AL and EBV-HLH, respectively, with high specificity (98.2% and 100%). Additionally, mediastinal LLNs in M-HLH were predominantly located in the retroinnominate space, whereas AL and EBV-HLH showed a predilection for the subcarinal space. This distribution difference may be related to the pathological nature of the diseases: subcarinal lymph nodes are more prone to hyperplasia due to antigen stimulation in infectious or inflammatory processes (e.g., EBV-HLH), while retroinnominate space involvement may be more frequently observed in malignant infiltration (e.g., lymphoma-associated M-HLH) (20.21). Similar findings were observed for axillary LLNs, with larger short diameters in M-HLH providing additional support for differential diagnosis. Pleural effusion was significantly less common in AL (14.0%) compared to M-HLH (60.9%) and EBV-HLH (67.3%). This difference may reflect the distinct pathogenetic mechanisms: pleural effusion in HLH (both M-HLH and EBV-HLH) is often secondary to systemic inflammation and increased vascular permeability, while AL is less likely to cause significant pleural involvement in the early stages (15, 20).

Hepatomegaly was highly prevalent in all three groups (≥91.4%), limiting its utility as a differentiating feature. However, WPS showed significant intergroup differences: the highest incidence was in EBV-HLH (78.8%), followed by M-HLH (54.5%), and the lowest in AL (15.5%). According to relevant literature, WPS in HLH is likely due to inflammatory cell infiltration into the hepatic interstitium rather than tumor cell infiltration, which may explain the higher incidence in HLH subtypes compared to AL (14, 21, 22). Splenomegaly was most common in EBV-HLH (94.2%), consistent with the role of the spleen as a major site of hemophagocytosis and immune activation in EBV-HLH (23). Ascites showed a similar distribution pattern to pleural effusion, with significantly lower incidence in AL, further supporting the role of systemic inflammation in HLH-related fluid accumulation (21, 23).

Intrarenal focal lesions (IFLs) of kidneys were rare in all groups, but particularly uncommon in EBV-HLH (1.9%). This finding suggests that if a child with suspected EBV-HLH presents with renal lesions on CT, coexisting diseases or complications should be excluded (23). Additionally, the absence of iliac fossa LLNs in EBV-HLH, compared to their presence in M-HLH and AL, provides another valuable differentiating clue.

The short diameter of abdominal LLNs was significantly larger in M-HLH, with cutoff values of 9.3 mm and 9.60 mm for distinguishing M-HLH from AL and EBV-HLH, respectively. These values, combined with the findings of mediastinal and axillary LLNs, highlight the importance of lymph node size measurement in CT imaging for differential diagnosis of these malignancies.

This study has several limitations. First, as a retrospective study, potential selection bias and confounding factors cannot be completely eliminated, which may affect the generalizability of the results. Second, the sample size, particularly for M-HLH (n=23), is relatively small, precluding multivariate analysis to adjust for potential confounders. Third, histopathological confirmation of organs such as the liver and kidneys was not obtained, which may lead to inadequacies and biases in the interpretation of imaging signs. Fourth, although imaging assessments were performed independently by two radiologists with consensus resolution, formal interobserver agreement statistics (e.g., Cohen’s kappa for categorical variables and intraclass correlation coefficient for continuous variables) were not calculated because the independent raw assessment records prior to consensus were not retained due to the retrospective nature of the study. This limits the quantitative assessment of measurement reproducibility. Fifth, the ROC-derived cutoff values for lymph node size are dataset-specific and lack external validation; thus, they require validation in independent cohorts before clinical application. Sixth, as this was a single-center study conducted at a tertiary referral center in Southwest China, the generalizability of our findings to other populations, healthcare settings, or geographic regions may be limited. External validation in independent, multicenter cohorts is needed before our conclusions can be broadly applied. Seventh, due to the extremely low clinical incidence of leukemia-associated hemophagocytic lymphohistiocytosis (AL-HLH) in the pediatric population, our cohort did not contain a sufficient number of AL-HLH cases to permit a separate subgroup analysis comparing AL-HLH with AL alone. Future multicenter studies are needed to accumulate adequate AL-HLH cases for further validation.

It is important to acknowledge that CT imaging should be considered only as an adjunctive tool in diagnostically challenging cases or when complications such as pulmonary or abdominal involvement are suspected. The imaging features described in this study should always be interpreted in conjunction with clinical and laboratory findings, and should not be used in isolation to diagnose or exclude HLH.

Thoracic and abdominal CT manifestations exhibit distinct characteristics among pediatric M-HLH, AL, and EBV-HLH presenting with acute pancytopenia. Key differentiating features include the incidence of pulmonary lesions, TBB, pleural effusion, WPS, splenomegaly, ascites, and IFLs, as well as the size and distribution of lymph nodes in the mediastinum, axilla, and abdomen. These imaging indicators, combined with clinical and laboratory data, may provide supportive information that could aid clinicians in the differential diagnostic process.

## Data Availability

The original contributions presented in the study are included in the article/**Supplementary Material**. Further inquiries can be directed to the corresponding authors.
