# Supplementary material for: Early imaging differentiation of children with acute hematologic malignancies that present in pancytopenia: a retrospective study
Source: Front Oncol. 2026 May 28;16:1775816. doi: 10.3389/fonc.2026.1775816 (PMC13253479; doi:10.3389/fonc.2026.1775816)
Supplement: Supplementary file 1 [file Table1.docx]

**Supplementary Table S1** CT imaging techniques used for the patients

|  | **Thorax** | **Abdomen** |
| --- | --- | --- |
| **Scanning technique** |  |  |
| Kv | 80 kV | 80 kV |
| mAs | 10-160 mA (automatic), with smart mA | 10-160 mA (automatic), with smart mA |
| Width of detector (mm) | 40 | 40 |
| Slice thickness (mm) | 5 | 5 |
| Pitch | 0.984 | 1.375 |
| Rotation time (s) | 0.6 | 0.6 |
| Interslice spacing (mm) | 5 | 5 |
| Reconstruction algorithm | Stnd | Stnd |
| Iterative algorithm | ASiR-V | ASiR-V |
| CTDIvol (mGy) | 1.5 | 4.2 |
| **Contrast enhancement** |  |  |
| Contrast agent | Omnipaque (350 mg/mL) | Omnipaque (350 mg/mL) |
| Dose of contrast agent (mL) | Body weight (kg) × 1.5 | Body weight (kg) × 1.5 |
| Injection rate (mL/s) | Dose/20 | Dose/20 |
| Delay time (s) | 30 | 45 |
